# Construction and validation of cell cycle-related prognostic genetic model for glioblastoma

**DOI:** 10.1097/MD.0000000000039205

**Published:** 2024-10-04

**Authors:** Runpeng Zhou, Kai Zhang, Tingting Dai, Zeshang Guo, Tian Li, Xinyu Hong

**Affiliations:** a Department of Neurosurgery, Pu’er People’s Hospital, Pu’er, China; b Department of Neurosurgery, Changzheng Hospital, Naval Medical University, Shanghai, China; c Department of Neurosurgery, The First Bethune Hospital of Jilin University, Changchun, China; d School of Basic Medicine, Fourth Military Medical University, Xi’an, China.

**Keywords:** glioblastoma, GSVA, nomogram, prognosis

## Abstract

Glioblastoma (GBM) is a common primary malignant brain tumor and the prognosis of these patients remains poor. Therefore, further understanding of cell cycle-related molecular mechanisms of GBM and identification of appropriate prognostic markers and therapeutic targets are key research imperatives. Based on RNA-seq expression datasets from The Cancer Genome Atlas database, prognosis-related biological processes in GBM were screened out. Gene Set Variation Analysis (GSVA), LASSO-COX, univariate and multivariate Cox regression analyses, Kaplan–Meier survival analysis, and Pearson correlation analysis were performed for constructing a predictive prognostic model. A total of 58 cell cycle-related genes were identified by GSVA and analysis of differential expression between GBM and control samples. By univariate Cox and LASSO regression analyses, 8 genes were identified as prognostic biomarkers in GBM. A nomogram with superior performance to predict the survival of GBM patients was established regarding risk score, cancer status, recurrence type, and mRNAsi. This study revealed the prognostic value of cell cycle-related genes in GBM. In addition, we constructed a reliable model for predicting the prognosis of GBM patients. Our findings reinforce the relationship between cell cycle and GBM and may help improve the prognostic assessment of patients with GBM. Our predictive prognostic model, based on independent prognostic factors, enables tailored treatment strategies for GBM patients. It is particularly useful for subgroups with uncertain prognosis or treatment challenges.

## 1. Introduction

Gliomas account for up to 30% to 40% of all brain tumors and 80% of all malignant brain tumors. Among them, glioblastoma is the most common and aggressive primary malignant brain tumor in adults, accounting for more than half of all malignant gliomas. Glioblastomas are characterized by high recurrence and mortality rates, and a low cure rate.^[1]^ The 5-year survival rate of patients with malignant glioma is < 30% for grade III (anaplastic astrocytoma) and 5% for grade IV (glioblastoma) tumors.^[2–4]^ Age, tumor grade, clinical treatment strategies, and the molecular phenotype of the tumor markedly affect the prognoses of patients with gliomas. The treatment efficacy for gliomas is suboptimal, and the prognosis of these patients is highly variable.^[5,6]^ The standard treatment for glioblastoma typically includes gross total resection surgery, focal radiotherapy targeting the tumor area, temozolomide chemotherapy, and a certain dose of radiotherapy as the standard treatment.^[7,8]^ Although these treatment modalities can extend patients’ survival to some extent, all glioblastomas eventually progress or recur.^[7]^ Existing prognostic models often rely on clinical pathological features, but they have certain limitations, such as low predictive accuracy and lack of personalized prediction.^[9]^Therefore, establishing an individualized prognostic scoring system based on important prognostic factors may facilitate the provision of individualized risk assessment and clinical treatment.

Gene Set Variation Analysis (GSVA), a gene set enrichment (GSE) method that estimates variation of pathway activity over a sample population in an unsupervised manner, can be used to assess the results of chipset gene enrichment. It evaluates the differences in the signal pathways by transforming the gene expression matrix among samples into gene sets expression matrix (signaling pathways) with the same biological function between samples. As a bioinformatics analysis method, GSVA can explain the causes of phenotypic differences according to the signal pathways.^[10]^ The foundation of the gene-rich integrated gene set (signaling pathway) was mainly derived from KEGG,^[11]^ BIOCARTA, and REACTOME,^[11,12]^ 3 general reference databases for signaling pathways. A large amount of biological information data, such as genome, metabolic pathways, and signaling pathways, is contained in above databases.^[13]^ The analysis and investigation of signal pathways and biological processes related to GBM are primarily based on KEGG in this study. In this study, GSVA could be utilized to compare differences in gene expression among different patients, aiding we in identifying changes in biological processes or pathways related to the disease. Specifically, GSVA analysis could be employed to determine whether gene sets associated with tumor development were significantly enriched in glioblastoma patients.

This study was based on the TCGA^[14]^ database and a combination of bioinformatics methods, GSVA analysis, and modeling analysis were used to identify key genes in relation to the differential function of GSVA in glioblastoma. The GSVA analysis helped us pinpoint specific genes that play a crucial role in the functional differences observed in glioblastoma. Subsequently, we employed LASSO Cox regression analysis to develop a risk score model, which integrated the expression levels of these key genes to predict patient outcomes. By utilizing these advanced computational methods, we were able to construct a comprehensive prognostic model. The accuracy of the model in predicting the prognosis of patients with glioblastoma was verified internally and externally.

## 2. Materials and methods

### 2.1. Data sources

**Figure 1.**
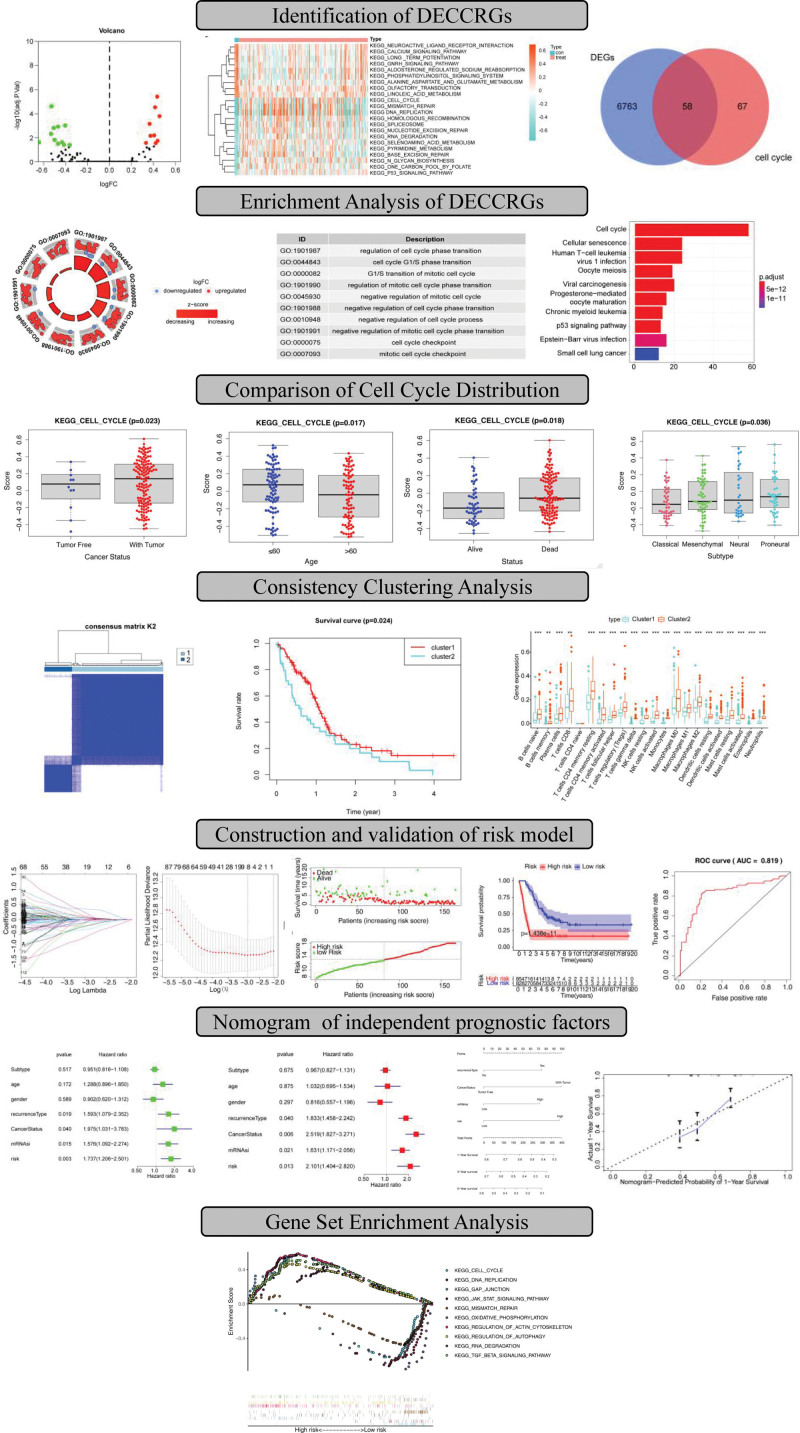
Flowchart.

Gene expression profiles and clinical information for the training cohort (https://portal.gdc.cancer.gov/projects/TCGA-GBM) of 166 patients with glioblastoma were collected from the Cancer Genome Atlas (TCGA) database (https://cancergenome.nih.gov).^[14]^ In the TCGA-GBM dataset, there were 104 male patients (62.7% of the total), 56 female patients (33.7% of the total), and 6 cases (3.6% of the total) where the gender was not reported. Concerning age at diagnosis, 9 patients were under 40 years old (5.4% of the total), 49 patients were aged between 40 and 60 (29.5% of the total), 71 patients were over 60 years old (42.8% of the total), and 37 cases had unreported age data (22.3% of the total). Regarding treatment status, 52 patients received treatment (31.3% of the total), 11 patients did not receive treatment (6.6% of the total), and treatment information was not reported for 103 cases (62% of the total).^[15]^ GSE13041 (https://www.ncbi.nlm.nih.gov/geo/query/acc.cgi?acc=GSE13041) and GSE43378 (https://www.ncbi.nlm.nih.gov/geo/query/acc.cgi) were downloaded from the Gene Expression Omnibus (GEO) database (https://www.ncbi.nlm.nih.gov/geo/) as the validation dataset. The flowchart of this study was shown in Figure 1

### 2.2. Identification of significantly differential pathways by GSVA

GSVA was performed to identify differential pathways using the GSVA package in R with default parameters. GSVA enhances the ability to detect subtle changes in pathway activity across sample populations.^[10]^ The default parameters in the GSVA package were chosen for their established effectiveness in pathway analysis and their widespread use in similar studies, ensuring consistency and comparability with existing literature.^[16,17]^ By identifying differentially expressed pathways, we could gain a deeper understanding of the molecular alterations associated with GBM progression and potentially uncover novel therapeutic targets.^[16]^ Consistent clustering of principal component analysis (PCA) and nonnegative matrix factorization (NMF) was verified to determine the correlation between cell cycle-related genes and GBM. DESeq2 R package was used to distinguish DEGs between 161 glioblastoma samples and 5 adjacent non-tumor samples using the threshold |log2(fold change)| > 1 and *P*-value < 0.05. The cell cycle-related genes were selected based on GSVA analysis, where we downloaded the pathway gene set KEGG_CELL_CYCLE from the Molecular Signatures Database (MSigDB) at https://www.gsea-msigdb.org/gsea/msigdb/. Differentially expressed cell cycle-related genes (DECCRGs) were obtained by overlapping DEGs with cell cycle-related genes from GSVA. DECCRGs were used for the subsequent analysis. GO^[18]^ and KEGG enrichment analysis of DECCRGs were performed using the clusterProfiler R package. This function analyses would help in understanding the biological functions and relevant pathways of DECCRGs in glioblastoma, providing insights into the potential roles and mechanisms of these genes in the pathogenesis of glioblastoma. In this analysis include the identification of key biological processes, molecular functions, and pathways that are dysregulated in glioblastoma, which can further our understanding of the disease at a molecular level.

### 2.3. Construction of the prognostic risk score

Univariate analysis was conducted with the COX regression model to investigate the prognostic values of cell cycle-related genes (*P* value < 0.05 and hazard ratio (HR) ≠ 1). Then, the lambda value of each candidate survival-associated gene was calculated by LASSO-COX analysis using the glmnet and survival packages in R. In this study, the model construction process involved using 10-fold cross-validation to determine the value of λ, and the λ value with the smallest partial likelihood deviance was selected as the optimal λ. Finally, the risk score was calculated using the following formula:


Risk score=ExpGene1×Coef1+ExpGene2×Coef2+ExpGene3×Coef3   (1)

where ExpGene is the expression level of the gene and Coef is the corresponding lambda value.^[19]^ For the TCGA data, the median value of risk scores in all patients for whom survival information was available was deemed as the cutoff. The same criterion was used to determine the cutoff value in the GEO database. The cutoff value was chosen as it represents a division point in the distribution of risk scores.^[19]^ Then, patients with GBM were assigned to the “high risk” or “low risk” group accordingly.

### 2.4. Correlation between prognosis and risk score

Kaplan–Meier survival curves were generated to illustrate the correlation between prognosis and risk score. Univariate and multivariate Cox regression analysis was conducted to identify independent risk factors for survival. In the univariate Cox analysis, variables including pathological subtype, age, gender, recurrence type, cancerstatus, mRNAsi, and risk score were incorporated. Subsequently, factors meeting the criteria of a *P*-value <0.05 and a HR different from 1 in the univariate Cox analysis were included in the multivariate Cox analysis.

### 2.5. Statistical analysis

The Kaplan–Meier method was used for survival analysis and the between-group differences were assessed using the log-rank test. The predictive accuracy of the risk model was determined by receiver operating characteristic (ROC) curve analysis. All data were analyzed using R (version 4.0.0). *P* values < 0.05 were considered indicative of statistical significance unless specified otherwise.

## 3. Results

### 3.1. Cell cycle pathway was highly correlated with clinicopathological features of GBM

**Figure 2.**
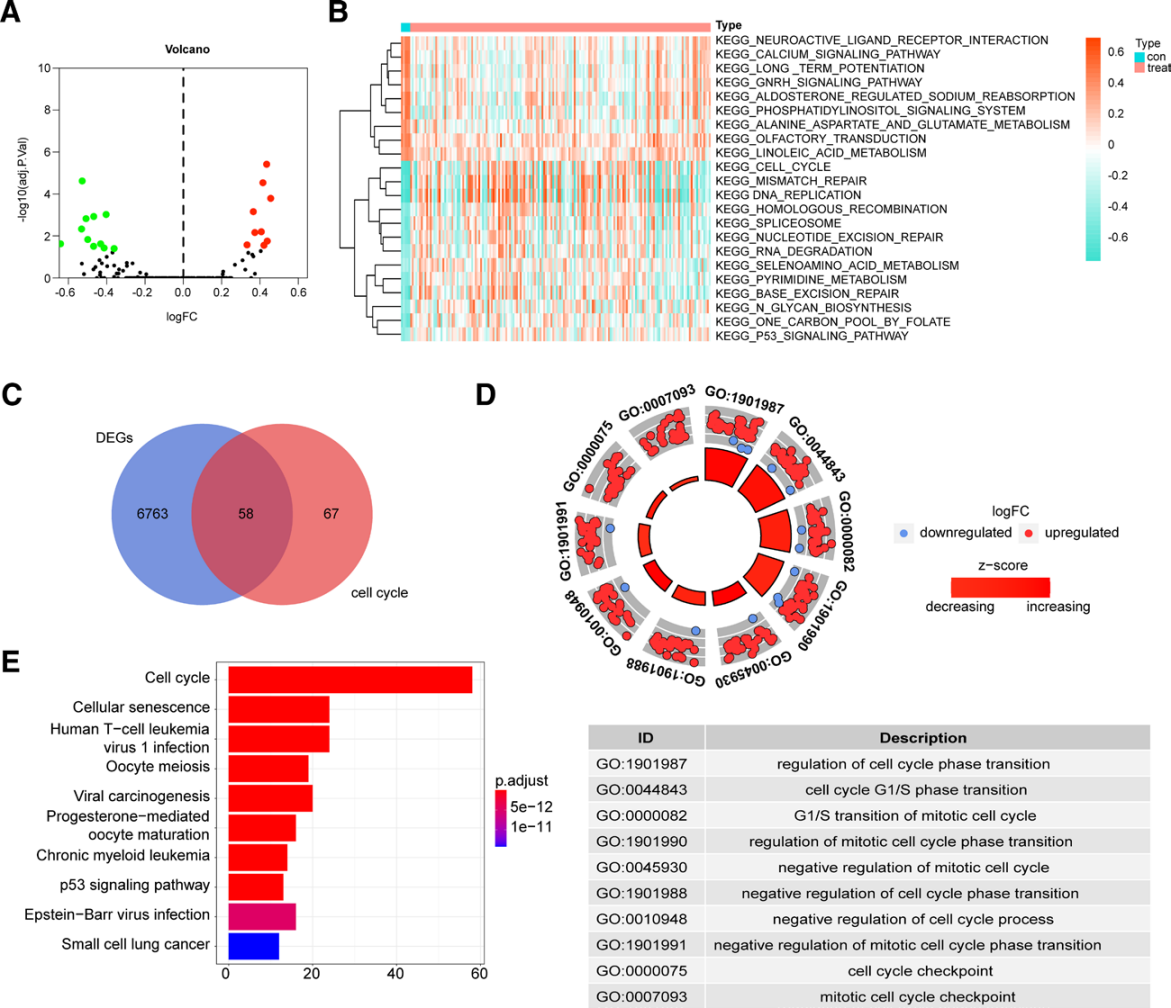
GSVA algorithm using the TCGA database. Volcano plot showing the differentially expressed pathways in GBM (A). Green dots represent significantly downregulated pathways and red dots represent significantly upregulated pathways in GBM with high expression. The heatmap (B) showing the expression level. (C) Venn diagram of overlapping 6821 DEGs and 125 cell cycle-related genes. (D, E) GO and KEGG functional enrichment analysis of DECCRGs.

To screen out the most significant differentially expressed pathways in GBM, enrichment scores of pathways were obtained by the GSVA algorithm in the TCGA database. The results identified that 20 pathways that were significantly different between glioblastoma samples and adjacent non-tumor samples (11 pathways were downregulated and 9 pathways were upregulated in glioblastoma) with log2FC > 1 and *P* value < 0.05, such as DNA replication were associated with resistance to GBM^[20]^ (Fig. 2A). The cell cycle pathway showed the most significant difference between GBM and control samples (Fig. 2B), indicating a crucial role of the cell cycle pathway in the genesis of GBM. A total of 58 cell cycle-related differential genes (DECCRGs) were obtained by overlapping 6821 DEGs with 125 cell cycle-related genes (Fig. 2C) and were used for the subsequent analyses. Functional enrichment analysis showed that the top GO terms were associated with cell cycle transition, such as regulation of cell cycle phase transition, cell cycle G1/S phase transition, G1/S transition of mitotic cell cycle, regulation of mitotic cell cycle phase transition, and negative regulation of mitotic cell cycle (Fig. 2D). Interestingly, apart from the cell cycle, we found that DECCRGs were also involved in infection, which has a complex interaction with the immune system, such as the human T-cell leukemia virus 1 infection pathway, viral carcinogenesis pathway, and p53 signaling pathway (Fig. 2E). Having been reported in the literature, the crucial role of the p53 signaling pathway in GBM is highlighted, with the inhibition of this pathway by SNRPG being shown to enhance the sensitivity of GBM cells to TMZ, thereby aiding in overcoming chemotherapy resistance.^[21]^ These pathways may play a role in the development or progression of GBM, and targeting them could offer potential therapeutic strategies for inhibiting GBM progression.

**Figure 3.**
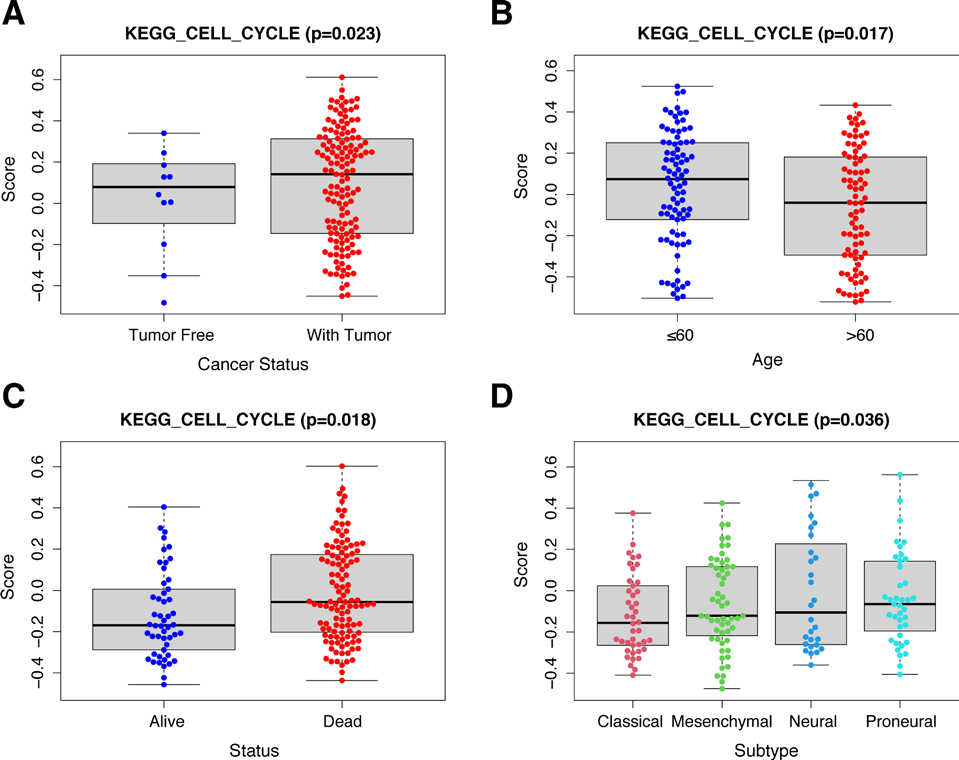
Cell cycle score were significantly different among groups stratified by cancer status (A), age (B), status (C), subtype (D).

In our study, we compared cell cycle scores across different clinicopathological features using appropriate statistical methods.^[22,23]^ Specifically, we applied ANOVA to compare cell cycle scores among groups stratified by cancer status, age, status, and subtype. post hoc multiple comparison corrections were also performed to control for false positives. By utilizing these statistical methods, we were able to identify significant differences in cell cycle scores across the stratified groups. Furthermore, we assessed the significance levels of these differences to underscore the impact of various clinicopathological features on cell cycle scores. In short, we compared cell cycle scores across different clinicopathological features and identified significant differences in the cell cycle scores among groups stratified by cancer status (Fig. 3A), age (Fig. 3B), status (Fig. 3C), and subtype (Fig. 3D). The higher cell cycle scores in the proneural subtype, as well as in tumor status, age less than or equal to 60 years, and death status, across these 4 clinical subgroups, indicate a significant impact on GBM progression. The higher cell cycle scores may reflect an increased rate of cell proliferation, which could be associated with the invasiveness and malignancy of the tumor.^[24]^ Therefore, the results suggest that the abnormal activation of the cell cycle may be an important factor in the progression of GBM, providing valuable clues for further research on the role of cell cycle regulation in the development and treatment of GBM.

**Figure 4.**
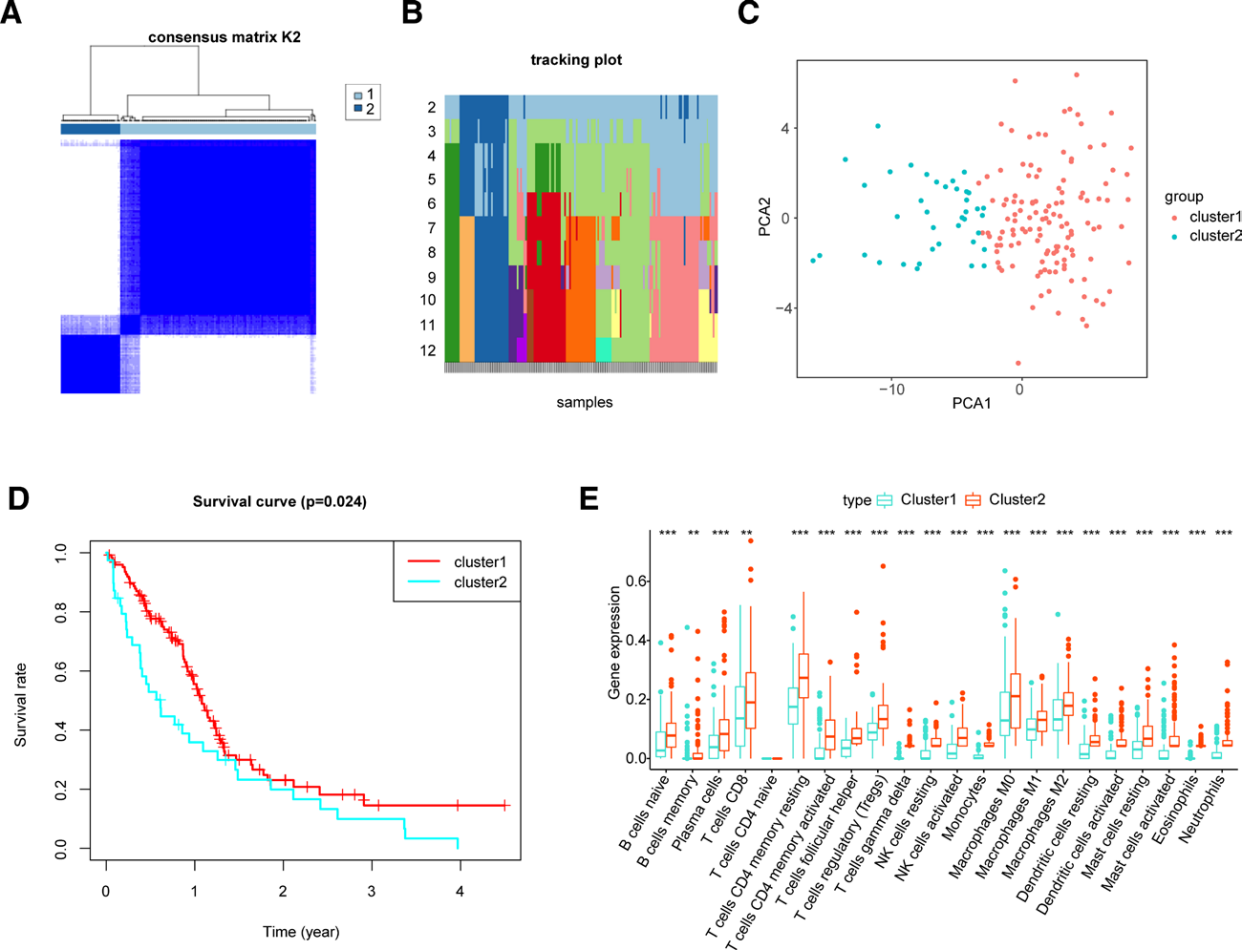
GBM patients were separated into 2 groups. (A) Consensus clustering matrix for *k* = 2, which was the optimal cluster number in the cohort. (B) Tracking plot for *k* = 2 to 12 in the samples. (C) Principal component analysis (PCA) plot of the samples. (D) Kaplan–Meier curves for cluster1 and cluster2. (E) Comparison of the gene expressions between different NMF subgroups in the training cohort. The gene expressions of 22 immune cells are displayed in boxplots.

Based on 125 cell cycle-related genes expression, when rank = 2, meaning when GBM patients were separated into 2 groups, NMF results revealed the improvement of categorization (Fig. 4A). The criteria included: consistent sample numbers in each group, a gradual and smooth rise in the cumulative distribution function curve, and stronger intragroup links compared to weaker inter-group links after clustering.^[23]^ The consistency distribution for *k* values ranging from 2 to 12 was displayed in the empirical cumulative distribution function plot. Based on the NMF rank survey, 2 subgroups were selected by the standard of rank = 2, named cluster1 and cluster2 (Fig. 4B). Furthermore, to validate the effectiveness of unsupervised clustering, the PCA could clearly demonstrate the distinctions between the 2 clusters. The PCA analysis showed that all samples were PC1 negative (Fig. 4C). Most cluster1 cases were PC1 positive, and the majority of cluster2 cases were PC2 positive. These results indicated significantly different spatial distribution of samples disaggregated by NMF grouping. In terms of clinical outcomes, comparisons between cluster1 and cluster2 showed significant differences in Kaplan–Meier survival analysis (Fig. 4D), suggesting that the DECCRGs were responsible for the prognostic differences. Given the individual immune cell scores, we further analyzed the differences in immune cell composition of various NMF subgroups in the training cohort and identified distinct immune cell composition between cluster1 and cluster2 (Fig. 4E).

### 3.2. Construction and validation of cell cycle-related risk score model in GBM

**Figure 5.**
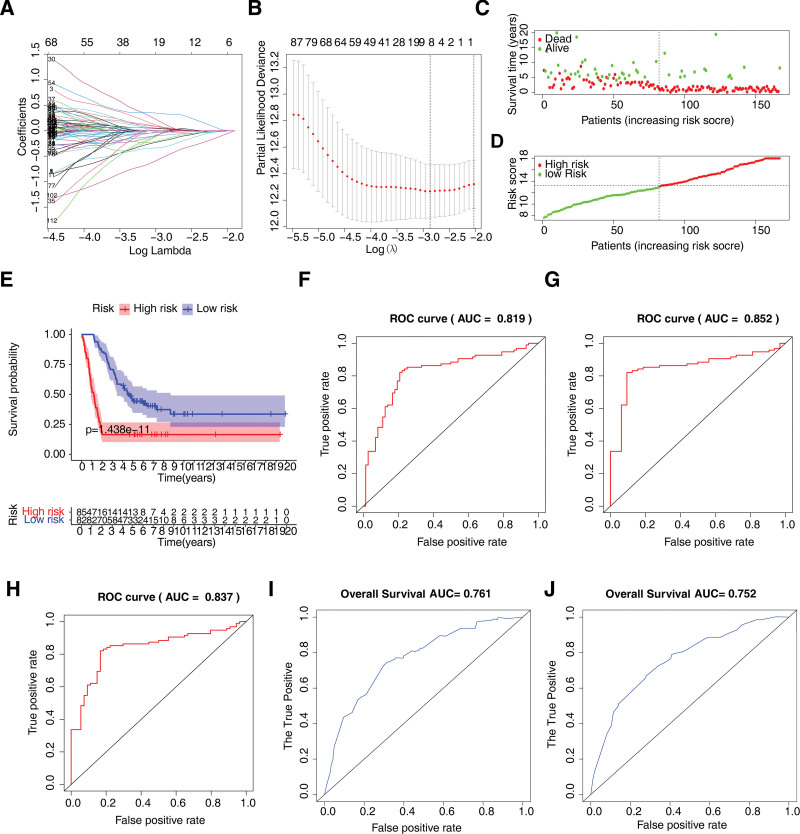
Eight DECCRGs were input into LASSO algorithm. (A) LASSO coefficient profiles of the 58 cell cycle-related differentially expressed genes. (B) Selection of optimal λ value in the LASSO model for glioma. (C) Distribution of risk score in the cohort. (D) Scatter plot of patients with different survival statuses. (E) Kaplan–Meier (K-M) survival analyses showing the survival difference between the low- and high-risk groups. (F, G, and H) ROC curves showing the predictive efficiency of the risk model in the 1-, 3-, and 5-year sets. (I and J) ROC curves showing the prediction efficiency of the risk model in the validation set.

Thereafter, the prognostic value of 58 DECCRGs in GBM was evaluated by univariate Cox and LASSO regression analyses. On univariate Cox regression analysis, *ANAPC4, CCNB1, CDC25B, CHEK1, PLK1, SMAD3, YWHAG,* and *YWHAZ* were identified as being significantly related to the survival of GBM patients. To obtain a more robust prognostic signature in this study, we utilized 10-fold cross-validation to determine the optimal value of λ in the LASSO algorithm, selecting the λ with the smallest partial likelihood deviation as the optimal λ. These 8 DECCRGs, namely ANAPC4, CCNB1, CDC25B, CHEK1, PLK1, SMAD3, YWHAG, and YWHAZ, were then identified through further stepwise selection (Fig. 5A and B) and defined as prognostic DECCRGs (Fig. 5A and B). The formula for calculating the risk score of each patient can be derived based on the LASSO regression coefficients. Then the risk score of each GBM patient in the training set was calculated as the expression of ANAPC4 * −0.082 + expression of CCNB1 * 0.015 + expression of CDC25B * −0.013 + expression of CHEK1 * 0.008 + expression of PLK1 * 0.115 + expression of SMAD3 * 0.048 + expression of YWHAG * 0.699 + expression of YWHAZ * 0.60. In clinical applications, the risk scoring model based on cell cycle-related genes can serve as a valuable tool for predicting the prognosis of GBM patients. Previous studies have combined single-factor Cox regression and LASSO to build a prognostic model related to copper death in GBM, guiding the prognosis and personalized treatment of GBM.^[25]^ A comparison between the performance of the scoring model and existing prognostic models for glioblastoma can provide insights into their potential advantages and clinical utility.

In comparison to existing prognostic models for glioblastoma, the risk scoring model may demonstrated superior predictive accuracy and discrimination. According to the median value of risk scores, the GBM patients in the TCGA training set were divided into high- and low-risk groups (Fig. 5C). Increase in the risk scores was associated with higher rate of death (Fig. 5D). The high-risk group had worse survival compared to the low-risk group (Fig. 5E). The area under the ROC curve (AUC) for 1-, 3-, and 5-year survival were 0.819, 0.852, and 0.837, respectively (Fig. 5F, G, and H), suggesting an excellent performance of the risk score model in predicting the prognosis of GBM patients. Furthermore, on testing the risk score model in GSE13041 and GSE43378 validation sets, the AUC values were 0.696 and 0.677, respectively, (Fig. 5I and J), demonstrating the reliability of the risk score model in predicting the survival of GBM patients.

### 3.3. Development of the cell cycle-related nomogram for GBM

**Figure 6.**
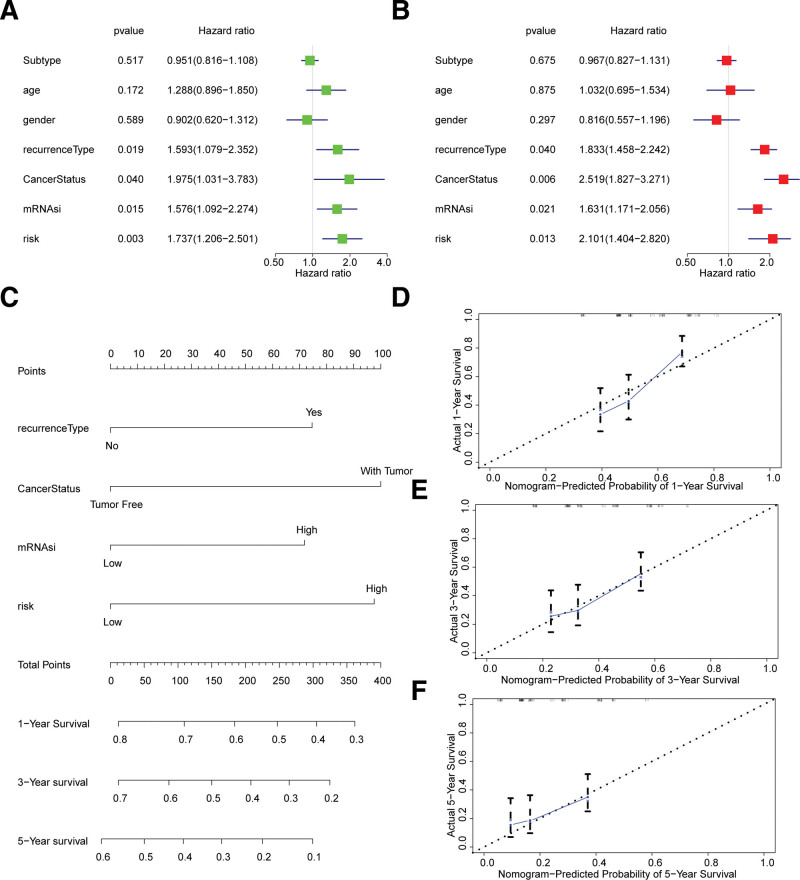
Prognostic value of risk score in GBM. (A and B) Univariate and multivariate Cox regression of risk score. (C, D, E, and F) Nomogram to predict the 1-, 2-, and 3-year overall survival rates of patients with GBM. The dashed diagonal line represents the ideal nomogram, and the blue lines represent the 1-, 2-, and 3-year observed nomograms.

Next, on multivariate analysis, the risk scores, mRNAsi, cancerstatus, and recurrencetype were identified as independent prognostic factors in GBM (Fig. 6A and B). Using these independent prognostic factors (risk scores, mRNAsi, cancerstatus, and recurrencetype), nomograms for predicting the 1-, 3-, and 5-year survival of GBM patients were established (Fig. 6C). Calibration curves showed that the predicted probability of overall survival approximated the actual overall survival (Fig. 6D and F), indicating good performance of the nomogram. Thus, this models could help doctors more accurately assess a patient’s risk profile, which was expected to improve patient survival and prognosis.^[26]^

### 3.4. Prognostic cell cycle-related genes may affect the progression of GBM by regulating cell proliferation

**Figure 7.**
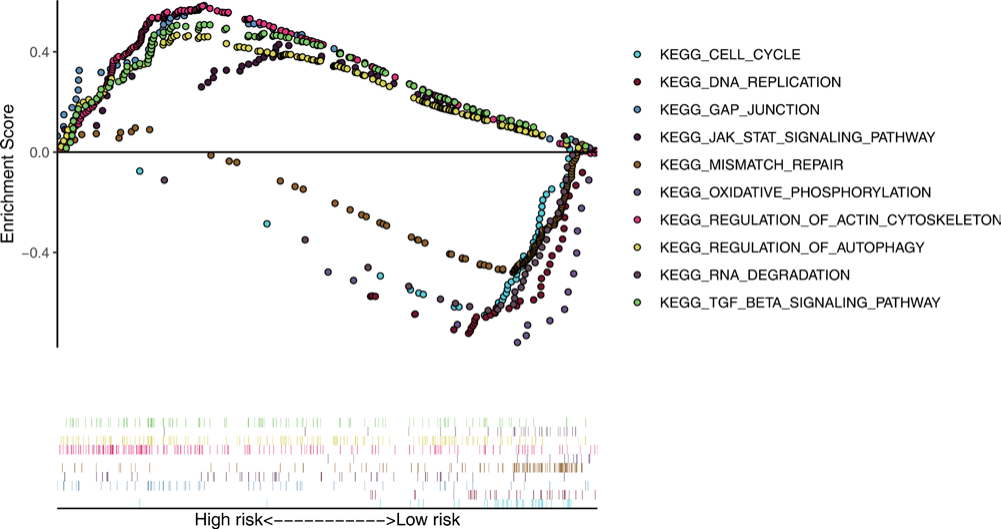
KEGG pathway enrichment analysis using GSEA. Cell cycle and DNA replication were significantly enriched in the high-risk group.

Next, we performed GSEA analysis to unravel the potential molecular mechanisms. The results showed significant enrichment of the KEGG pathways participating in the cell proliferation, such as cell cycle, DNA replication and JAK/STAT (Fig. 7), in the high-risk group. For instance, inhibition of JAK or STAT phosphorylation decreases antiapoptotic protein levels, leading to apoptosis in GBM cells.^[27]^ Studies have found that curcumin inhibits STAT activation and downstream target genes involved in cell proliferation, migration, and invasion, reducing GBM cell migration, invasion, and proliferation.^[28]^ These results suggested that prognostic cell cycle-related genes may regulate the development and progression of GBM via cell proliferation. Therefore, further exploration of how these cell cycle-related genes affect the growth and progression of glioblastoma would contribute to a deeper understanding of the underlying molecular mechanisms.

## 4. Discussion

The purpose of this study was to identify key genes that affect the prognosis of glioblastoma patients, construct a prognostic model combined with GSVA to predict the survival, and explore the potential regulatory mechanisms in glioma.

Neoplasms remain the main killer worldwide.^[29–34]^ In this study, we identified 58 DECCRGs in GBM compared to control tissues. These genes were principally involved in the biological process and signal pathway of cell cycle and DNA replication. The key cellular and molecular mechanisms involved in tumorigenesis are disorder of cell cycle regulation, uncontrolled growth, and reduction of apoptosis. Cell cycle has been shown to play a decisive role in the occurrence and development of GBM.^[35,36]^ In both breast cancer (BRCA) and GBM, key roles were played in cell cycle regulation, with lncRNAs TUG1, LINC01355, CASC9, and FOXC2-AS1 impacting cell proliferation and apoptosis in breast cancer, while cell cycle arrest leaded to reduced proliferation and subsequent cell death in GBM, revealing specific differences in cell cycle regulation between BRCA and GBM.^[37,38]^

Thereafter, we assessed the prognostic value of 58 DECCRGs in GBM using univariate Cox and LASSO regression analyses. On univariate Cox regression analysis, *ANAPC4, CCNB1, CDC25B, CHEK1, PLK1, SMAD3, YWHAG*, and *YWHAZ* were identified as being significantly related to the survival of GBM patients. Based on the results, a risk score model and nomogram for predicting survival of GBM patients were established. These 8 genes are well-established cell cycle factors, which are involved in the regulation of M phase of mitotic cell cycle, cell cycle checkpoint, and cell arrest, etc. It has been found by multiple studies that CCNB1 regulates the occurrence of cancer, such as hepatocellular carcinoma, pancreatic cancer, and lung adenocarcinoma, by mediating the cell cycle.^[39–41]^ In a previous study, the expression of *CCNB1* showed a negative correlation with the therapeutic efficacy of temozolomide (TMZ) in low-grade gliomas.^[42]^ Some studies have reported that CCNB1, as a biomarker of GBM, overexpression accelerates mitosis and promotes the proliferation and invasion of GBM tumor cells.^[43]^ Therefore, it was speculated that this gene may promote the development of GBM through the regulation of the cell cycle process. In recent years, it has been found that CHEK1 may play a role in colorectal cancer by affecting cell cycle regulation, and it is closely related to the development and progression of CRC.^[44]^ *CHEK1* is an important therapeutic target in gliomas. Inhibition of *CHEK1* may increase the radiosensitivity of gliomas.^[45,46]^ Research reports indicate that CHEK1 plays a crucial role in promoting proliferation and inducing radioresistance in GBM, while its inhibition can enhance apoptosis, suggesting a potential therapeutic target in GBM.^[47]^ In addition, studies have reported that the cell cycle-related gene PLK1 is associated with poor prognosis of cancer, and in addition, the gene may promote cell proliferation and migration of different types of cancer.^[48,49]^ Down-regulation of *PLK1* expression was found to enhance the inhibitory effect of TMZ on glioma stem cells.^[50]^ Additionally, dual inhibition of PLK1 and STAT3 co-regulates the MYC pathway in GBM, leading to reduced MYC expression, enhanced cell invasion and apoptosis, and tumor suppression, suggesting a potential therapeutic strategy for targeting GBM.^[51]^These genes, particularly CCNB1, CHEK1, and PLK1, have been implicated in the development and progression of cancer, including gliomas and colorectal cancer. Their potential as therapeutic targets and their impact on treatment outcomes suggest their importance in the prognosis of GBM patients. To summarize, we believe that there is adequate rationale for selection of these 8 cell cycle markers.

Our research results indicated a negative correlation between risk score and prognosis, suggesting that patients with higher risk scores might have poorer prognosis. Furthermore, the K-M curve based on independent prognostic factors could effectively predict patients’ survival at different time points, providing new theoretical basis for personalized treatment or better improvement of patients’ survival. In addition, the nomogram constructed based on independent prognostic factors can also predict the survival rate of GBM patients at different times. However, due to limitations in the sample resources of the dataset, validation in datasets from more diverse sources might be necessary to ensure the applicability of this model.

Finally, we analyzed the potential mechanism by which these 8 prognostic cell cycle markers regulate GBM. Subsequent GSEA analysis demonstrated that the high-risk group was significantly enriched in the biological processes and signal pathways involved in cell proliferation, metastasis, and invasion. This suggests that patients of high-risk were more likely to exhibit increased cellular growth, metastatic potential, and invasive characteristics, which may contribute to a poorer prognosis or more aggressive disease behavior. Some studies have reported that by promoting cell cycle arrest, it might be possible to slow down or inhibit the proliferation of GBM cells, thereby potentially impacting the aggressiveness and growth of high-risk GBM tumors.^[52]^ Therefore, we speculate that these 8 genes regulate metastasis and deterioration through the above biological processes and signal pathways, and ultimately affect the prognosis of patients with GBM.

Some limitations of this study should be acknowledged. The retrospective nature of the study may have induced an element of selection bias. Second, several other factors affect the postoperative outcomes, such as preoperative examination, preoperative comorbidities, operative time, and occurrence of serious postoperative complications, none of which were included in this study. Further studies involving larger datasets are required to establish a more accurate prognostic model for GBM patients. Furthermore, further research with larger datasets and potentially incorporating experimental studies could help in the development of a more robust and accurate prognostic model for GBM patients, which could aid in the improvement of treatment strategies and patient outcomes. Finally, exploration of the therapeutic potential of identified genes, or investigation of the applicability of research models in personalized medicine will provide a roadmap for the advancement of this field in the future.

In summary, this study identified 8 cell cycle-related genes related to prognosis in GBM, explored the potential molecular mechanism, and established a prognostic model for GBM patients.

## Acknowledgments

The manuscript has been edited and proofread by Medjaden Inc., and was supported by the Medjaden Academy & Research Foundation for Young Scientists (Grant No. MJA202306050).

## Author contributions

**Conceptualization:** Zeshang Guo, Xinyu Hong.

**Data curation:** Runpeng Zhou, Kai Zhang.

**Formal analysis:** Runpeng Zhou, Tingting Dai.

**Investigation:** Runpeng Zhou, Kai Zhang, Tian Li.

**Methodology:** Runpeng Zhou.

**Project administration:** Tian Li.

**Resources:** Runpeng Zhou.

**Software:** Runpeng Zhou, Tingting Dai.

**Supervision:** Zeshang Guo.

**Validation:** Runpeng Zhou.

**Visualization:** Zeshang Guo.

**Writing – original draft:** Runpeng Zhou.

**Writing – review & editing:** Kai Zhang, Tingting Dai, Zeshang Guo, Tian Li.
